# Associations of Socioeconomic Status and Healthy Lifestyle With Incidence of Dyslipidemia: A Prospective Chinese Governmental Employee Cohort Study

**DOI:** 10.3389/fpubh.2022.878126

**Published:** 2022-06-09

**Authors:** Ling Li, Feiyun Ouyang, Jun He, Dan Qiu, Dan Luo, Shuiyuan Xiao

**Affiliations:** Department of Social Medicine and Health Management, Xiangya School of Public Health, Central South University, Changsha, China

**Keywords:** socioeconomic status, lifestyles, dyslipidemia, incidence, China

## Abstract

**Objective:**

The purpose of the study was to test whether primary lifestyles mediate associations of SES with incidence of dyslipidemia and to explore interaction relations of lifestyles and SES with incidence of dyslipidemia.

**Methods:**

We included 9,901 individuals at baseline from January 2018 to November 2019, and incidence data were updated to 31 December 2020. Dyslipidemia was defined as total cholesterol (TC) 6.2 mmol/L TC ≥ or triglycerides (TG) ≥2.3 mmol/L or low-density lipoprotein cholesterol (LDL-C) ≥4.1 mmol/L or high-density lipoprotein cholesterol (HDL-C) <1.0 mmol/L; or physician diagnosed dyslipidemia or lipid-lowering drugs use. Lifestyles, socioeconomic factors, and personal characteristics were collected by a questionnaire. A latent class analysis based on education, family income, and occupational position was used to assess the SES. Lifestyle score was calculated using cigarette smoking, alcohol consumption, physical activity, and diet. Cox proportional hazard models and multivariate analyses were used to explore the associations. The mediation effect was evaluated using bootstrap method.

**Results:**

Participant mean age was 36.5 years (SD = 0.11). The cumulative incidence of dyslipidemia was 11.0% over a mean follow-up of 13.4 months. Compared with participants of high SES, those with low SES had higher risk of incidence of dyslipidemia [hazard ratio 1.32, 95% confidence interval (CI): 1.01–1.73], after adjusting for lifestyle scores and other covariates. The proportion mediated by lifestyles was 5.41% (95%CI: 4.17–7.11). A significant additive interaction was found between lifestyles and SES, whereas association between lifestyle and incidence of dyslipidemia was stronger among those of high SES. Additionally, individuals with low SES and no or one healthy lifestyle behavior had a higher risk of developing dyslipidemia than those with high SES and 3 or 4 healthy lifestyles.

**Conclusion:**

Unhealthy lifestyles play a small moderating role in socioeconomic inequity in incidence of dyslipidemia among Chinese governmental employees, suggesting that promoting healthy lifestyles alone may not significantly reduce socioeconomic inequalities in health, and measures to address other social determinants of health should also be considered alongside.

## Introduction

Globally, dyslipidemia, generally characterized by an elevated level of blood lipids, including low-density lipoprotein cholesterol (LDL-C), total cholesterol (TC), triglycerides (TG), or a decreased level of high-density lipoprotein cholesterol (HDL-C), has been considered as an independent preventable risk factor of cardiovascular disease (CVD) (1–4). For instance, for every reduction of 39 mg per deciliter (1.0 mmol/L) in LDL-C level, rates in cardiovascular events and all-cause mortality reduce to 22 and 10%, respectively (2, 5). However, the prevalence of dyslipidemia in Chinese adults was up to 40.40% in 2012, which was significantly higher than that in 2002 (6). Although the prevalence of dyslipidemia is still lower in China than those in western country, the dyslipidemia control rate of the former was seven-folds lower than the latter (7), generating that the burden of CVD has increased significantly.

Socioeconomic status (SES) has been commonly defined as the combination of education, income level, and occupation (8). SES was related to the differences in morbidity and mortality, but results were conflicting due to the differences in cultural factors and national development (9, 10). Over the last few decades, although China's economic reforms have triggered unprecedented economic growth and social resources have grown as well, there is an increasing wealth gap between individuals with high and low economic levels, causing a large inequity in medical service utilization (11, 12). The impacts of these inequalities have been more pronounced during the COVID-19 pandemic, with the greatest influence on socially disadvantaged groups (13). Thus, immediate efforts are essential to reduce socioeconomic inequities in health and increase the resilience of population.

Numerous epidemiological studies have established that lifestyle factors are the main risk factors associated with dyslipidemia (14, 15). Lifestyle factors are generally considered to be the mediators between SES and health (10). However, to our knowledge, if there exists and how much lifestyles mediate the association between SES and incidence of dyslipidemia is still not clear. Additionally, previous studies were prone to use single socioeconomic indicator (e.g., income, education, and occupation) to partly reflect SES; hence, it is necessary to generate an indicator, including primary aspects of SES. Additionally, lifestyles are mutually connected and limited studies conducted lifestyle score to evaluate its effect on the socioeconomic inequities in dyslipidemia. Based on the literature, both SES (low SES) (16, 17) and unhealthy lifestyles (e.g., less physical activity, irrational diet, etc.) (18, 19) are associated with the risk of dyslipidemia. Besides, the evidence on the effect of the interaction and joint links between SES and lifestyles on incidence of dyslipidemia is insufficient. Thus, potential interactions between socioeconomic status and lifestyle on the incidence of dyslipidemia were assessed in our study. We hypothesize that the associations between SES and incidence of dyslipidemia will be differed by lifestyles, age, sex, or BMI variable level in different directions and magnitudes.

The aims of this study are as follows: (1) to assess whether SES is associated with the incidence of dyslipidemia in Chinese governmental employee population; and (2) if such association exists, to explore the mediating role of potentially modifiable lifestyle behaviors in it.

## Methods

### Study Population

**Figure 1:**
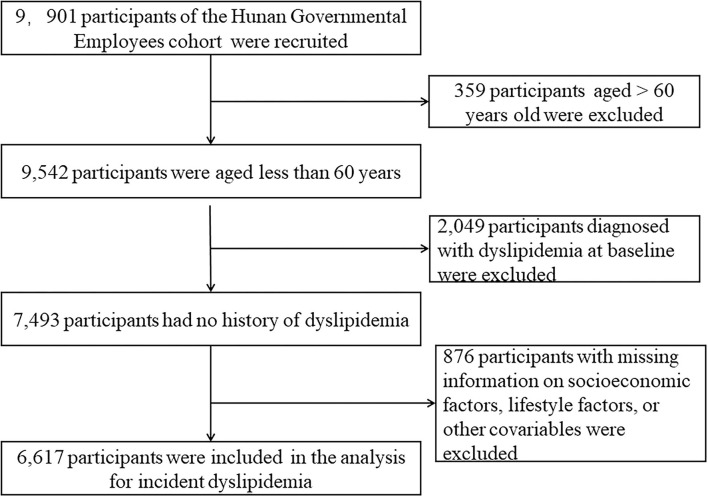
Flowchart of the study.

This study was based on the Cohort study on Chronic Diseases of Governmental Employees, which was carried out in five major cities (Changsha, Zhuzhou, Huaihua, Xiangtan, and Changde) of Hunan Province, China. Detailed information on study design of this cohort has been described in our previous study (20). Briefly, a multistage sampling design was carried out to obtain a representative sample from January 2018 to November 2019, and incidence data were updated to 31 December 2020. First, five cities in Hunan Province were selected based on the levels of economic development and geographic location. Second, several institutions were randomly sampled from the government-run institutions that volunteered to participate in the study. Third, in each sampled institution, all the employees were selected to take part in our study using cluster sampling. After obtaining informed consent, eligible participants were asked to complete a digital self-reported questionnaire. Accordingly, this study included 9,901 participants at baseline. Those aged over 60 years were 359, and those diagnosed with dyslipidemia were 2,049 at baseline. Those with missing information on SES factors, lifestyles, and other covariates were excluded from the analysis (*n* = 876). Overall, 6,617 participants were eligible for the longitudinal analyses (Figure 1).

### Assessment of SES

Baseline self-reported education level, family income level, and occupational position were used to measure SES, and each factor was divided into three levels (low, medium, and high). Total family income was obtained through a digital self-reported questionnaire platform, which was applied in our previous study (20), and we grouped participants: low (≤ ¥50,000), middle (¥50,000–29,999), and high (≥¥300,000) according to the urban average annual wages of staff and workers on the job in Hunan Province, China [from Hunan Statistical Yearbook 2019 (21)]. Education was categorized into less than high school diploma, high school graduate or equivalent, and college or above. For the education grouping, we refer to the standards of the Japanese Civil Service Cohort due to its' special population as those in our study (22). A professional title, which represents one's qualifications and level in a certain professional skill in China, was divided into high- (senior professional technician, or manager at deputy division level or division level or above), medium- (intermediate professional technician, or manager at deputy section level or section level), and low- (primary professional technician, or grassroots staff/clerk) levels in this study. SES index was assessed using latent class analysis (23) based on family income, education level, and occupational position. A total of three latent classes were identified, which, respectively, represented a high, medium, and low SES according to the item-response probabilities (refer to the Supplementary Method).

### Assessment of Lifestyle Factors and Covariates

We assessed lifestyle factors at baseline with reference to questionnaires applied in previous large prospective studies (24, 25). We constructed a healthy lifestyle score, including cigarette smoking, alcohol consumption, physical activity, and diet, based on the earlier evidence of these factors' contribution to socioeconomic mortality differences or because of their potential role as a risk factor for mortality, especially from cardiovascular diseases (10, 24). All lifestyle factors were obtained through structured questionnaires. Never smoking was confirmed as a healthy level, which was defined in the questionnaire as smoking fewer than 100 cigarettes in life. A healthy alcohol consumption level was defined as frequency no more than one time a week. For leisure time physical activity, the pattern (jogging, bicycling, swimming, etc.), frequency (1–2 times, 3–5 times, and >5 times a week), and duration (<30 min, 30–60 min, or >60 min per time) were reported. Meanwhile, monthly metabolic equivalent hours of leisure time physical activity were calculated (Supplementary Method), according to Compendium of Physical Activities (26). To harmonize the data, we further classified the participants into thirds and defined the top third as a healthy level of physical activity. We evaluated dietary quality using a more recent dietary recommendation for cardiovascular health and combining with traditional Chinese eating habits, which considered adequate consumption of whole grains, fresh fruit, fresh vegetables, fish, shellfish, dairy products, and limited consumption of processed meats and sugar-sweetened beverages. A healthy diet was defined as meeting at least four items of the recommendations (Supplementary Table S1). For the above healthy lifestyles, we scored 1 for a healthy level and 0 for none. Therefore, the healthy lifestyle score was the total of the points ranging from 0 to 4, with higher scores showing healthier lifestyles.

Covariates were obtained through questionnaires, including age, sex, marital status, history of hypertension, diabetes, CVD, or cancer; and history of chronic bronchitis, emphysema, or chronic obstructive pulmonary disease (COPD) and family history of above common chronic diseases. Bodyweight and height were measured at baseline, with BMI calculated as weight (kg)/[height (m)^2^]. Sleep quality was evaluated using items of the Pittsburgh sleep quality index (PSQI) (27). Sleep quality was classified into good, fair, and bad. A healthy sleep quality was defined as a good status of sleep quality.

### Outcome Ascertainment

Blood samples were collected at 07:30–10:00 after a fasting period of 12 h. Fasting plasma TC, TG LDL-C, and HDL-C were measured at regular physical examination by trained medical staff using a Chemistry system Autoanalyzer in the Medicine Laboratory Department of each study sites at baseline and follow-up. Information on dyslipidemia history and lipid-lowering drugs use was collected from questionnaires at baseline and follow-up. Participants were defined as dyslipidemia at the annual physical examination if they met one of the following standards: (1) according to the Chinese guidelines on the prevention and treatment of dyslipidemia in adults (2016) (6): TC ≥6.2 mmol/L or TG ≥2.3 mmol/L or LDL-C ≥4.1 mmol/L or HDL-C <1.0 mmol/L; (2) self-reported physician diagnosed dyslipidemia or using lipid-lowering drugs for previously diagnosed dyslipidemia.

### Statistical Analysis

Baseline characteristics were described across different levels of SES, and differences among groups were tested by analysis of variance for continuous variables, and Pearson chi-squared test or Fisher's exact for categorical variables. We used Cox proportional hazard regression models to estimate the hazard ratios (HRs) and 95% confidence intervals (CIs) of incidence of dyslipidemia associated with SES and lifestyle score. The proportional hazard assumption was examined by follow-up time, and we found no significant deviation from the assumption. The parameters of dyslipidemia among different SES were compared using multivariate analyses. Age, sex, marital status, chronic diseases history (diabetes, hypertension, CVD, cancer, chronic bronchitis, emphysema, or COPD), and family history of common chronic diseases (diabetes, hypertension, and CVD) were selected as the important covariates to be adjusted in our models, based on the previous research (16).

We used the bootstrap method to identify the mediation effects of lifestyles for the association between SES and incidence of dyslipidemia (28, 29). Bootstrap could test the indirect effect without making any assumptions about the shape of the sampling distribution of the indirect effect, which is already widely used and recommended in mediation analysis, due to its more power and better type I error control than the Sobel test and the causal steps approach (30). It allows direct testing of the product of the coefficients, with the null hypothesis of no mediation, H0: a^*^b = 0, by seeing whether 0 is within the estimated CI (*a* denotes the coefficient of independent variable on mediator; *b* denotes the coefficient of mediator on dependent variable, controlling for independent). Bootstrapping is a computationally intensive procedure that involves sampling of the rows of the data with replacement to build a new sample of size *n* from the original sample. In the new “resample,” a^*^b is estimated. This process is repeated *R* times (ideally, *R* is in the thousands) to build a bootstrap distribution of the indirect effect. A 95% confidence interval for the indirect effect using the percentile method is defined by two values of $\hat{\text{a}}\hat{\text{b}}$ in the bootstrap distribution of *R* estimates that define the 2.5th (the lower limit) and the 97.5th (the upper limit) percentiles of the distribution of estimates taken from the B resamples. As with other confidence interval-based methods of inference, an indirect effect can be said to be different from zero if the confidence interval excludes zero. In our mediation analysis, to add precision to the percent attenuation, a bias-corrected bootstrap method with 2,000 resamples was used to obtain 95% CIs of the direct and indirect effects. Age, sex, marital status, baseline BMI, history common chronic diseases, and family history of common chronic diseases were adjusted in mediation models as covariates. Coefficients are presented in standardized form, using standardized coefficients as indices of effect. A statistically significant mediation effect is observed when the 95%CI does not include zero. The proportion mediated (PM) was used to evaluate the effect size of the mediation analysis.

We used stratified analysis by latent class of SES to evaluate associations of the lifestyle score with incidence of dyslipidemia. As only 78 (1.2%) and 609 (9.2%) individuals had 0 and 4 points of healthy lifestyle score, we combined participants with scores of 0 and 1, and those with scores of 3 and 4. Participants with unhealthy lifestyle behaviors (score of 0 or 1) were analyzed as a reference group in this analysis. Relative excess risk due to interaction (RERI) and corresponding 95%CI was measured in additive interaction model, calculated using the coefficients, corresponding standard errors, and covariance matrix (31). Moreover, participants were divided into nine groups based on the SES (low, medium, and high) and healthy lifestyle score (0 or 1, 2, and 3 or 4 points) to assess the joint association, and hazard ratios of incidence of dyslipidemia were estimated in different groups compared with those with high SES and 3 or 4 points of healthy lifestyle score.

To verify the robustness of the results, four models were constructed in the sensitivity analysis based on the previous researches (24, 32). In model 1, age, sex, and marital status were directly included as covariates. In model 2, prevalent comorbidities (including hypertension, diabetes, cardiovascular disease, cancer, and chronic bronchitis, emphysema, or chronic obstructive pulmonary disorder) and family history of the diseases (including hypertension, diabetes, and cardiovascular disease) were included. In model 3, BMI was additionally incorporated. In model 4, the healthy lifestyle score was additionally included to evaluate the HRs of incidence of dyslipidemia associated with SES from models with and without the hypothesized mediator. Besides, we constructed a lifestyle score, including baseline sleep quality in sensitivity analysis. Meanwhile, participants with major chronic diseases at study baseline could largely influence the lipid metabolism. Therefore, we conducted a sensitivity analysis by excluding participants who have major chronic diseases at baseline. In addition, we used interaction terms to test whether there is effect modification of age, sex, or BMI on association between SES and incidence of dyslipidemia. The hazard ratio with its 95% confidence interval of the interaction terms was the measure of interaction. Then, we tested the robustness and potential variations in different subgroups stratified by sex (men and women), age groups (<45 years, and ≥45 years), and BMI (18.5–24.9, <18.5, and ≥25.0).

All analyses were performed using Stata version 13.0 (StataCorp LP, College Station, Texas, USA) and R 3.6.2 with “ggplot2” and “epiR” packages. A two-sided *p*-values <0.05 were considered as being of statistical significance.

## Results

### Population Characteristics

Table 1 shows baseline characteristics of participants. A total of 6,617 participants were included, with an average age of 36.5 years [standard deviation (SD): 0.11] and 25.7% men. Among them, 1,346 (20.3) were of high SES, 4,595 (69.4%) of medium SES, and 676 (10.2%) of low SES by LCA method. Those of low SES were more likely to be women, not married, and less educated, and to have low income and occupational position, and a higher prevalence of family history of CVD than those of high SES. Unhealthy levels of leisure-time physical activity, diet, and sleep quality were more prevalent among participants of low SES. Those excluded from this study owing to missing information were younger, of high SES, and more likely to be women (Supplementary Table S2).

**Table 1:** Baseline characteristics of participants according to socioeconomic status (SES)^*^.

**Characteristics**	**Total population** **(*n* = 6,617)**	**High SES^†^** **(*n* = 1,346)**	**Medium SES^†^** **(*n* = 4,595)**	**Low SES^†^** **(*n* = 676)**	*p*-Value*
Mean age (mean, SD)	36.5 (0.11)	43.3 (0.23)	34.7 (0.12)	35.0 (0.40)	**<0.001**
Men	1,702 (25.7)	519 (38.6)	1,017 (22.1)	166 (24.6)	**<0.001**
Married	5,213 (78.8)	1,243 (92.3)	3,536 (77.0)	434 (64.2)	**<0.001**
Household income					
High	491 (12.0)	489 (36.3)	0 (0.0)	2 (0.3)	<0.001
Medium	5,333 (80.6)	857 (63.7)	4,366 (95.0)	110 (16.3)	
Low	793 (7.4)	0 (0.0)	229 (5.0)	564 (83.4)	
Education					
College or above	6,373 (96.3)	1,346 (100.0)	4,550 (99.0)	477 (70.6)	**<0.001**
High School or equivalent	195 (3.0)	0 (0.0)	42 (0.9)	153 (22.6)	
Less than high school	49 (0.7)	0 (0.0)	3 (0.1)	46 (6.8)	
Occupational position					
High or above	1,103 (16.7)	1,018 (75.6)	77 (1.7)	8 (1.2)	**<0.001**
Medium	2,443 (36.9)	199 (14.8)	2,244 (48.8)	0 (0.0)	
Low	3,071 (46.4)	129 (9.6)	2,274 (49.5)	668 (98.8)	
Never smoking	6,092 (92.1)	1,194 (88.7)	4,291 (93.4)	607 (89.8)	0.461
No heavy alcohol consumption	6,336 (95.8)	1,242 (92.3)	4,455 (97.0)	639 (94.5)	0.061
Top third LIPA	2,212 (33.4)	629 (46.7)	1,333 (29.0)	161 (23.8)	**<0.001**
Healthy diet^#^	1,536 (23.2)	570 (42.3)	1,460 (31.8)	170 (25.1)	**<0.001**
Healthy sleep quality^&^	2,746 (41.5)	674 (50.1)	1,787 (38.9)	285 (42.2)	**<0.001**
BMI (mean, SD)	23.3 (0.07)	23.9 (0.15)	23.1 (0.09)	23.2 (0.23)	0.922
Self-reported comorbidities					
Hypertension	126 (1.9)	51 (3.8)	51 (1.1)	24 (3.6)	0.789
Diabetes	51 (0.8)	20 (1.5)	20 (0.4)	11 (1.6)	0.807
CVD	23 (0.4)	7 (0.5)	12 (0.3)	4 (0.6)	0.140
Cancer	69 (1.0)	17 (1.3)	44 (1.0)	8 (1.2)	0.897
Chronic bronchitis or COPD	23 (0.4)	4 (0.3)	14 (0.3)	5 (0.7)	0.173
Family history of diseases					
Hypertension	2,272 (34.3)	465 (34.5)	1,597 (34.8)	210 (31.1)	0.117
Diabetes	814 (12.3)	153 (11.4)	579 (12.6)	82 (12.1)	0.614
CVD	576 (8.7)	95 (7.1)	412 (9.0)	69 (10.2)	**0.015**

*SD, standard deviation; BMI, body mass index; COPD, chronic obstructive pulmonary disease; CVD, cardiovascular disease; LTPA, leisure time physical activity.*

*SES was generated through latent class analysis using information on education, family income, and occupational position.*

^*^Continuous variables were expressed as mean (95% confidence interval), and categorical variables were expressed as number (percentage). p-Values were calculated using analysis of variance for continuous variables, and Pearson chi-squared test or Fisher's exact for categorical variables.

*Less than ¥50 000, ¥50 000 to 299 999, and ¥300,000 or more household income represented the high, medium, and low family income levels, respectively.*

*^#^Healthy diet denoted ideal intakes of≥4 dietary components for cardiovascular health.*

*^&^Healthy sleep quality was defined as a good status of sleep quality. Significant difference is highlighted by bold font (p <0.05)*.

### Associations of SES With Incident Dyslipidemia and Mediation Proportion of Socioeconomic Inequity in Morbidity Attributed to Lifestyles

The cumulative incidence of dyslipidemia was 11.0% (727/6,617) during a mean follow-up of 13.4 months. After adjusting for lifestyle score and other covariates (including age, sex, marital status, BMI, and history of comorbidities), the hazards ratio (HR) was 1.32 [95% confidence interval (CI): 1.01–1.73] when individuals with low SES were compared with those of high SES (Table 2). There were monotonically increasing trends for SES expressed as high, medium, and low groups (*p* <0.05) (Table 2). In model 3, the hazard ratio without adjustment for lifestyle score was larger (HR = 1.37, 95% CI: 1.04–1.79). Healthy lifestyles were associated with lower risks of incidence of dyslipidemia (HR = 0.90, 95% CI: 0.82–0.99), and the healthier the lifestyles, the greater the reduction in morbidity. Notably, we found that SES was indirectly associated with incidence of dyslipidemia through lifestyles (β = 0.001, 95% CI: 0.0002–0.003), and the proportion mediated by the lifestyle score was 5.41% (95%CI: 4.17–7.11%, *p*-value = 0.007) for incidence of dyslipidemia, when low SES was compared with high SES (Table 2).

**Table 2:** Associations of socioeconomic status with incidence of dyslipidemia and mediation proportion of socioeconomic inequity in health attributed to lifestyle.

**Analysis**	**Hazard ratio**	**95%CI**	*p-*Value*
**Model 1**			
High SES	1 (Reference)		
Medium SES	1.22	1.01–1.48	0.038
Low SES	1.34	1.02–1.75	0.034
**Model 2**			
High SES	1 (Reference)		
Medium SES	1.23	1.02–1.49	0.033
Low SES	1.36	1.03–1.78	0.027
**Model 3**			
High SES	1 (Reference)		
Medium SES	1.24	1.02–1.50	0.031
Low SES	1.37	1.04–1.79	0.024
**Model 4**			
High SES	1 (Reference)		
Medium SES	1.22	1.01–1.47	0.045
Low SES	1.32	1.01–1.73	0.047
**Mediation proportion (%, 95%CI)** ^†^	5.41	4.17–7.11	0.007

*SES, socioeconomic status; CI, confidence interval.*

*Model 1 adjusted for age, sex, marital status.*

*Model 2 adjusted for model1 + prevalent comorbidities (including hypertension, diabetes, cardiovascular disease, cancer, and chronic bronchitis, emphysema, or chronic obstructive pulmonary disorder) and family history of the diseases (including hypertension, diabetes, and cardiovascular disease).*

*Model 3 adjusted for model 2 + BMI.*

*Model 4 adjusted for model 3 + healthy lifestyle score.*

*Trend chi-square was used to test trending on Hazard ratios between high SES, medium SES, and low SES in four models (all p <0.05).

*Bias-corrected percentile method was presented based on 2,000 bootstrap samples*.

Supplementary Table S3 shows the association with the parameters of dyslipidemia and SES. After adjusted by lifestyle factors and other covariates, SES was positively associated with serum TG, and an increasing level of TG in low SES group was found than that in high SES group (standardized regression coefficient = 0. 052, *p-*value <0.001). The mediation proportion by lifestyles on above relationship was 10.3% (95%CI: 5.4–17.1, *p-*value = 0.001). There were no differences of the mean levels of TC, LDL-C, and HDL-C between low and high SES groups (all *p*-value > 0.05).

### Interaction and Joint Associations of Lifestyles and SES With Incident Dyslipidemia

**Figure 2:**
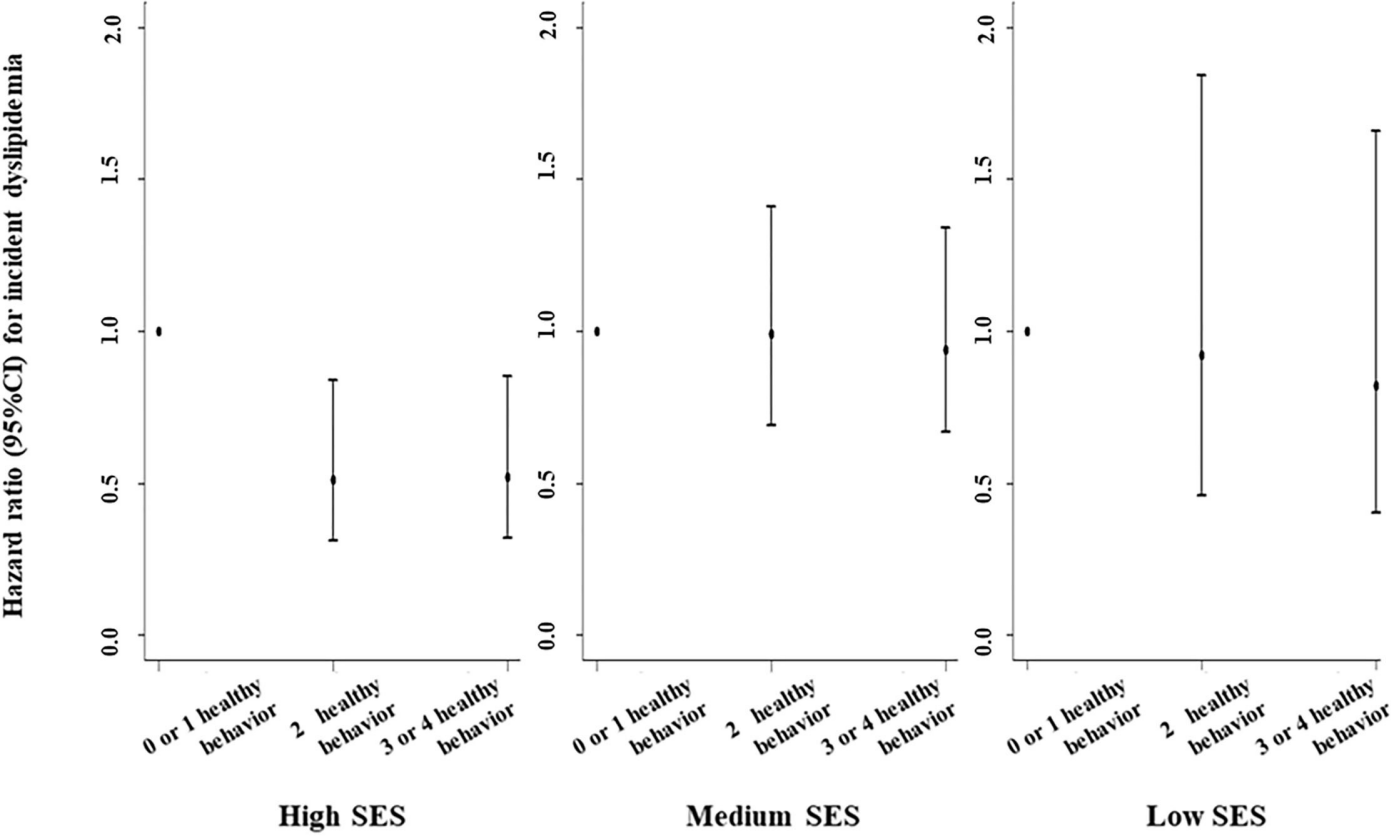
Associations of healthy lifestyle score with incidence of dyslipidemia by socioeconomic status (SES). Hazard ratios were adjusted for age, sex, marital status, self-reported comorbidities (including history of hypertension, diabetes, CVD, cancer, chronic bronchitis, emphysema, or chronic obstructive pulmonary disease), family history of diseases (including hypertension, diabetes, and CVD), and body mass index. Additive interaction was observed between lifestyle score and High SES on incidence of dyslipidemia (*p* for interaction was 0.029).

**Figure 3:**
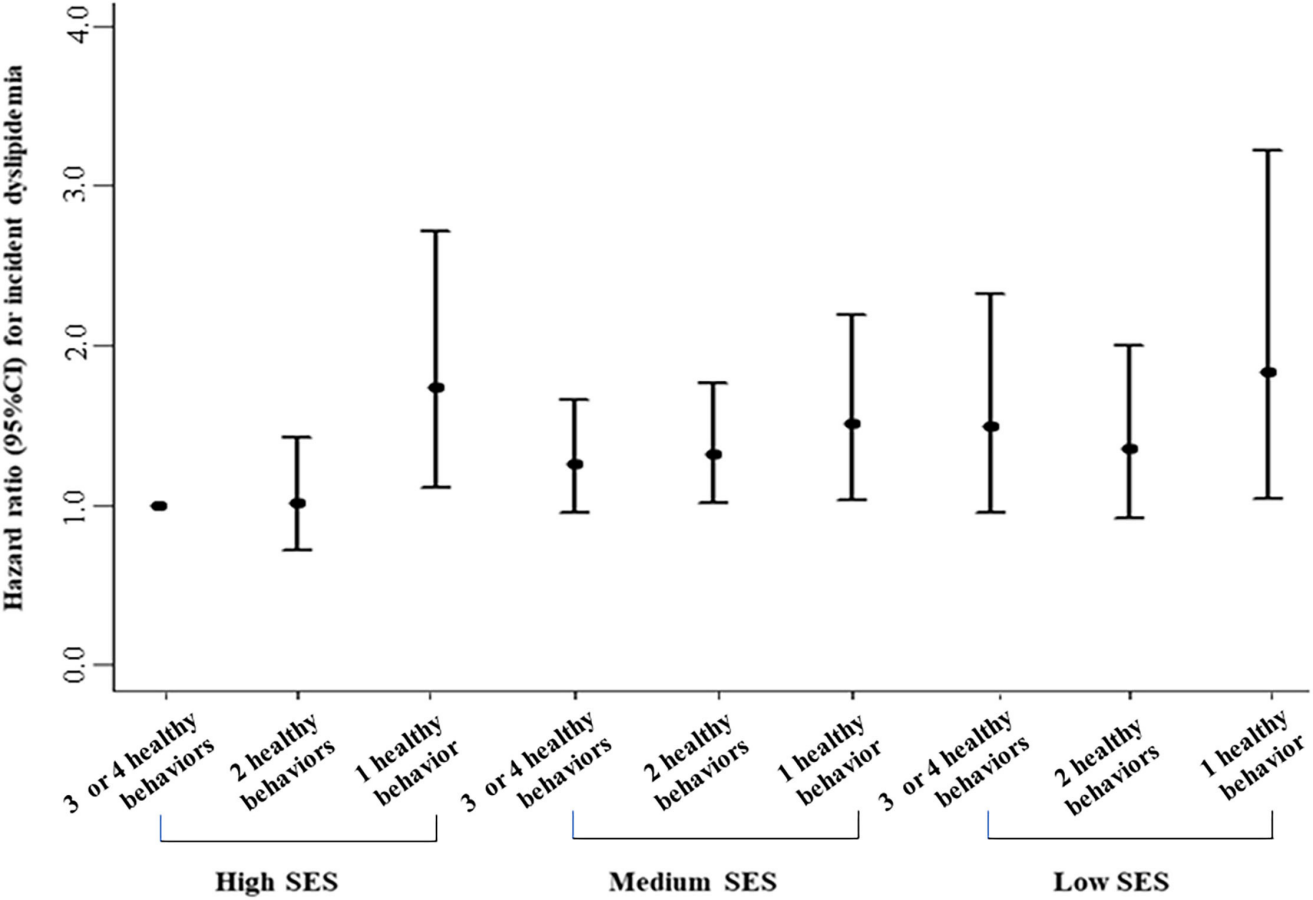
Joint associations of healthy lifestyle score and socioeconomic status with incidence of dyslipidemia. Hazard ratios were adjusted for age, sex, marital status, self-reported comorbidities (including history of hypertension, diabetes, CVD, cancer, chronic bronchitis, emphysema, or chronic obstructive pulmonary disease), family history of diseases (including hypertension, diabetes, and CVD), and body mass index.

A positively additive interaction was observed between lifestyle score and SES on incidence of dyslipidemia (RERI = 0.89, 95%CI: 0.59–1.20; Figure 2). Healthier lifestyle scores were associated with lower risks of incidence of dyslipidemia among individuals with a different SES, whereas the associations were stronger among those of a high SES (Figure 2). For example, the HR for those with 3 or 4 healthy lifestyle factors compared with no or one healthy lifestyle factor for incidence of dyslipidemia was 0.52 (0.32–0.85) among individuals with high SES. Figure 3 revealed the joint association of SES and lifestyles score on the incidence of dyslipidemia, and the HR for adults with low SES and no or one healthy lifestyle factor was 1.83 (1.04–3.22), when compared to those with high SES and three or four healthy lifestyle factors.

### Socioeconomic Inequity and Lifestyle in Incident Dyslipidemia by Sensitivity and Subgroup Analysis

In sensitivity analyses, we constructed several models and found that the results remained similar in all sensitivity analyses (Table 2 and Supplementary Table S4). We found that all the interaction terms were significant, indicating that age, gender, and BMI were the effect modification factors of association between SES and dyslipidemia (Supplementary Table S5). Then, we conducted subgroup analysis. Supplementary Table S6 shows the results stratified by sex, age group, and BMI groups, which were not materially changed with those of the main analyses. For instance, the socioeconomic inequity in incidence of dyslipidemia was stronger in men (HR = 1.48, 95%CI: 1.01–2.17) than in women, and in older (HR = 1.50, 95%CI: 1.02–2.30) than younger adults. Compared with normal BMI adults, overweight or obesity ones demonstrated a significantly greater inequity (HR = 1.54, 95%CI: 1.01–2.37). The proportions of socioeconomic inequity in morbidity mediated by lifestyles were all lower and similar to those of the main analyses (Supplementary Table S6).

## Discussion

In this longitudinal study, the cumulative incidence of dyslipidemia was 11.0%. Notably, low SES was associated with higher risk of incidence of dyslipidemia, and approximately 5.41% of the association were mediated by lifestyle scores. A significant additive interaction was found between lifestyle scores and SES on incidence, and the association was stronger among those of high SES. The highest risk of incidence of dyslipidemia was discovered in those of low SES and with the least healthy lifestyle behavior.

Socioeconomic inequality is a powerful independent predictor of CVD development and adverse outcomes (33–35). However, the impact on socioeconomic inequality in incidence of dyslipidemia is still not clear. Previous studies have confirmed that SES was related to the prevalence of dyslipidemia in various population. People with low SES were more prone to dyslipidemia than those with high SES (18, 19). Also, single socioeconomic factors have been extensively tested, whereas there were limited researches on the overall SES of individual (36, 37). Notably, in this study, we created an overall SES index based on education, family income, and occupational position, to represent SES level. Our findings verified the socioeconomic differences in incidence of dyslipidemia and extended the results to dyslipidemia parameters. Therefore, there is an urgent need to explore the possible ways to reduce socioeconomic inequalities in dyslipidemia which is urgently needed.

Results for the associations between SES and individual parameters of dyslipidemia are also debatable. A previous study in the US suggested that SES was negatively associated with serum TG levels (35), but a survey conducted in Switzerland revealed that lower education attainment was related to high TG level (38). Meanwhile, the inconsistent links between SES and other three parameters were also found in middle- and low-income countries (39, 40). In our study, we discovered that SES was positively associated with serum TG, and an increasing level of TG in low SES group was found than that in high SES group, which has shown similar results with a 10-year follow-up study in urban India (39), while inconsistent with another research (41). The reasons for the inconsistent results were confounded, but the differences in assessment of SES and the definition of population characteristics may provide us with possible clues. Therefore, there is an urgent to improve the socioeconomic inequality in blood lipid levels among Chinese government employees, especially for those with higher TG concentration and lower SES, because they may subject to a greater risk of coronary heart disease.

Numerous researches have showed the contribution of health-related behaviors to socioeconomic inequity in CVD (10, 42). About 20%−30% of socioeconomic inequalities in health outcomes can be explained by lifestyle, but there are differences between studies (36), resulting in impossibility to make a firm conclusion. The reasons for the discrepant contribution of lifestyles to socioeconomic disparities in health are complex and may be on the cultural differences between countries (43), demographic characteristics of the participants (e.g., age, gender, and health status) (44), differences in the SES measures, lifestyles and health outcomes assessed, and methodological differences in the evaluations of the contribution of lifestyles (45, 46). Lifestyles are interconnected (47), and a single or limited number of lifestyles was investigated in previous studies, which were not enough to comprehensively measure its impact on health, and few studies considered overall lifestyles when exploring the contributions (48, 49). In our study, hence, we created a composite index consisting of primary lifestyle factors (including cigarette smoking, alcohol consumption, physical activity, and diet) and found about 5.41% of the association between SES and incidence of dyslipidemia was explained by this index in Chinese governmental employees. The low mediation proportion suggested that for significantly reducing socioeconomic inequalities in health, other social determinants of health are necessarily considered alongside the promotion of healthy lifestyles. Here are some of the social determinants that are often cited as contributing to SES-health association, including lifestyle factors, environmental exposures, and psychosocial processes associated with stress exposure (8). Virtually every health behavior, including the four lifestyle variables considered in our study and others (e.g., sleep, sedentary behavior, etc.), are patterned by socioeconomic status. In this study, we found that unhealthy sleep quality was more prevalent among participants of low SES, which was consistent with the previous study (50). Although we constructed a lifestyle score, including baseline sleep quality in the sensitivity analysis, the results remained similar, suggesting that other unmeasured lifestyles may play a role, such as sedentary behavior (17, 51). Existing study shows SES-related health effects of social environments (e.g., isolation and the lack of engagement in social networks) may be even more important than those of physical environments (8). It also revealed that low SES population are susceptible to more environmental hazards and have access to fewer resources to reduce their influences. Exposure to stress is also a pathway. Previous study has found that chronic stress, such as negative cognitive-emotional factors (e.g., depression, anxiety), contributed to the associations of SES with lipids (52). To the best of our knowledge, our findings provided an unprecedented basis for exploring more evidence on the risk of incidence of dyslipidemia in the future, as so far, there is no such study conducted in governmental employee population.

Our study also indicated that healthy lifestyle scores were associated with lower risks of incidence of dyslipidemia in governmental employees, and the healthier the lifestyles, the greater the reduction in morbidity. In addition, significant interactions were observed, and the associations of healthy lifestyles with incidence of dyslipidemia were stronger among adults with high SES, which was inconsistent with the results in previous studies on associations between SES and lifestyles with other health outcomes (24, 32). In the context of the development of Public Institutions in China (53), public workers with higher SES (e.g., a higher occupational level) are mostly male and older, as this study showed (refer to Table 1), so they were more vulnerable to unhealthy lifestyles and the incidence of disease than those of low SES. The mechanisms underlying the interaction between SES and lifestyles on incidence of dyslipidemia have not yet been elucidated. A total of two possible explanations are proposed. First, attitudes and views on unhealthy lifestyle interventions for the prevention of disease may be one possible mechanism for this interaction between SES and lifestyles. For example, many people with low socioeconomic status felt that healthy lifestyles had less or no influence on their future health or disease onset, because it is largely predestined (54). Changing behaviors felt like a major hurdle for those with low SES, especially when unhealthy lifestyles had become a long-standing habit. So, they maintain a higher rate of unhealthy lifestyles, ultimately increasing the risk of disease. Second, the availability and relative cost of healthier foods, such as fresh fruits and vegetables, vary considerably across communities that vary by SES. Although described as a personal behavior, one's ability to eat a healthy diet and to exercise is affected by resources available to the person. Low SES communities often lack supermarkets and recreational facilities, resulting that the residents are more prone to consume and produce less fresh and be sedentary lifestyles (51, 55). Interactions have allowed increasingly more detailed and nuanced examination of the realities of the social patterning of health. In addition, we even found an association between SES and incidence of dyslipidemia among men, and 3.8% of relationship was explained by lifestyles, but not among women. A prior study of Japanese adults showed that lower household expenditures were related to a variety of cardiovascular risk factors, but not significantly with dyslipidemia (low HDL-C levels or high TG) in women (56). Another study conducted in the US revealed a weaker association between lifestyles and health outcome among men with low incomes, whereas not among women (57). This difference could be because smoking, heavy alcohol consumption, or physical inactivity behaviors might be more prevalent in men of low SES (20). Inconsistent definitions of SES and lifestyles also explain the part of the problem.

Moreover, obesity has always been a hot topic, and there is SES gradient (58). The growing obesity epidemic, with its relations with type 2 diabetes and coronary heart disease, is more acute among lower SES populations (59, 60). In this study, we included baseline BMI (18.5–24.9.2 vs. ≥25.0 kg/m^2^) in a subgroup analysis and found that those individuals with overweight or obesity were more prone to be a stronger association between low SES and incidence of dyslipidemia than those with normal BMI. Obesity is associated with diet- and exercise-related behaviors that control the balance of energy intake and expenditure (58). In our study, we found a slight proportion (3.8) of mediator by lifestyles on the association between SES and incidence of dyslipidemia in obesity groups, although it was not statistically different. These findings suggest that we should also take unhealthy behaviors change into account, especially in the obese group with low SES, when preventing and screening for the incidence of dyslipidemia, thereby preventing the development of cardiovascular disease.

The main strengths of this study are the large samples from established representative Chinese governmental employee cohort. Furthermore, a composite index of SES as well as healthy lifestyle score was created to assess the associations of SES and lifestyles with the incidence of dyslipidemia. In addition, we performed the joint and stratified analyses with sufficient statistical power, and we used sensitivity analyses to imply the robustness of the results. However, there were still some potential limitations. First, many variables were self-reported and only evaluated at a point in time, and thus, recall or evaluation biases were inevitable. If under-reporting of dyslipidemia information in the low SES group exits, the socioeconomic inequity in health outcome might not be accurately estimated. Besides, the lifestyle was only assessed by questionnaire at baseline without considering the effect of its change on the outcome. Future studies with repeated measurements will be necessary. This questionnaire may be not a standard assessment, despite we referred it to previous studies; therefore, to support the application of this questionnaire of lifestyles in such programs exploring the contribution of lifestyle to socioeconomic inequalities in health, it is important to first establish its construct validity. Additionally, we did not examine other possible lifestyles (e.g., sitting/sedentary behaviors) as the mediators of the SES-dyslipidemia association and cannot comment on the relative importance of health behaviors in relation to other mediators. Additionally, although SES was evaluated as a composite index (including income, education, and occupation) in our study, it cannot sufficiently capture SES because other additional SES aspects (wealth, residence, living environment, community resources, social support, etc.) were not considered. Second, participants in our study were from Hunan Province, China, although the sample of this special population is considered large, the results of the study may not be generalizable to other regions of China. Thus, future multiprovince or multinational monitoring studies are necessary to confirm our findings. Third, the follow-up duration is relatively short, and participants diagnosed during the study period were likely to have severe illness at baseline. SES and lifestyles might be influenced by health conditions. In this study, we adjusted the self-report comorbidities in main analysis to obtain robust results. Fourth, participants excluded from this study owing to missing information were younger, of high SES, and more likely to be women, which would lead to selection bias. Finally, although we controlled for key covariates in our Cox and mediation analysis, residual confounding by variables that are unknown or not included in the analysis may contribute to an overestimation of the role of lifestyles in the association between SES and incidence of dyslipidemia.

## Conclusion

In this large Chinese Governmental Employee Cohort, low SES was found to be significantly associated with higher risks of incidence of dyslipidemia, and the association was slightly mediated by lifestyle behaviors. Thus, for significantly reducing socioeconomic inequalities in dyslipidemia, other social determinants of health are considered alongside the promotion of healthy lifestyles. Notably, those with disadvantaged SES and unhealthy lifestyles had the highest risks of incidence, which emphasizes the importance of lifestyle modification to reduce the burden of disease for governmental employees, especially those with low SES in China.

## Data Availability Statement

The original contributions presented in the study are included in the article/Supplementary Material, further inquiries can be directed to the corresponding author.

## Ethics Statement

The studies involving human participants were reviewed and approved by The Ethics Committee of Xiangya School of Public Health, Central South University, China (No. XYGW-2016-10). The patients/participants provided their written informed consent to participate in this study.

## Author Contributions

LL was responsible for the study design, data analyses and interpretation, and manuscript writing and revision. FO, JH, and DQ were responsible for the data acquisition, interpretation, and manuscript revision. LL and DL were responsible for the manuscript revision. SX was responsible for the study conceptualization, data acquisition and interpretation, and manuscript revision. All of the authors approved the final content of this manuscript.

## Funding

This work was supported by the Ministry of Science and Technology of China (grant no. 2016YFC0900802).

## Conflict of Interest

The authors declare that the research was conducted in the absence of any commercial or financial relationships that could be construed as a potential conflict of interest.

## Publisher's Note

All claims expressed in this article are solely those of the authors and do not necessarily represent those of their affiliated organizations, or those of the publisher, the editors and the reviewers. Any product that may be evaluated in this article, or claim that may be made by its manufacturer, is not guaranteed or endorsed by the publisher.
