# Supplementary material for: Associations of Socioeconomic Status and Healthy Lifestyle With Incidence of Dyslipidemia: A Prospective Chinese Governmental Employee Cohort Study
Source: Front Public Health. 2022 Jun 9;10:878126. doi: 10.3389/fpubh.2022.878126 (PMC9218108; doi:10.3389/fpubh.2022.878126)
Supplement: Supplementary file 1 [file Data_Sheet_1.docx]

**Supplementary method**

***Assessment of socioeconomic status using latent class analysis***

Family income level, education level and occupational position reflect different aspects of socioeconomic status (SES), thus we used these three parameters to generate an overall SES parameter. Latent class analyses with different numbers of latent classes were conducted to select a reasonable model. The maximum absolute deviation between the parameter estimates in two successive iterations of the estimation procedure was set to 0.000001, which meant iteration would terminate when the difference between the parameter estimates in two successive iterations was less than 0.000001. Akaike information criterion (AIC), Bayesian information criterion (BIC), and likelihood ratio statistic G2 were used for the model selection. Item-response probability was a posterior probability and was used for defining latent classes.

We reported information on models with five or fewer latent classes. The following figure shows that G2 statistics, AIC, and BIC all continued to go down as more latent classes were added. However, the decrease leveled off after the three-latent-class solution. Thus, the three-latent-class solution was the best in terms of the uncertainty of posterior classification.

**Figure for supplementary method.** G2 statistics, AIC, and BIC in models with different numbers of latent classes.

**Table 1 for supplementary method.** Item-response probabilities in models with three to five latent classes*.

| **Item** | **Latent class 1** | **Latent class 2** | **Latent class 3** |
| --- | --- | --- | --- |
| Less than high school | 0.05 | <0.01 | <0.01 |
| High school or equivalent | 0.15 | <0.01 | 0.02 |
| College or university degree | **0.80** | **1.00** | **0.98** |
| Low house income | **0.52** | 0.01 | 0.09 |
| Medium house income | 0.47 | 0.25 | **0.91** |
| High house income | 0.01 | **0.74** | <0.01 |
| Low occupational position | **0.94** | 0.25 | **0.47** |
| Medium occupational position | <0.01 | **0.42** | **0.42** |
| High or above occupational position | 0.06 | **0.33** | 0.11 |

* The maximal item-response probabilities for each latent class were marked in bold.

Additionally, we evaluated the characteristics of three latent class in model. For the three-latent-class solution, latent class 3 was characterized by high-level family income, education, and occupational position, which could be defined as “high SES”; latent class 2 was characterized by medium-level family income, high education, and medium-low-level occupational position, which could be defined as “medium SES”; latent class 1 was characterized by low-level family income, occupational position, and high-level education, as well as low-level education, which could be defined as “low SES”.

***Assessment of physical activity***

We defined the metabolic equivalent scores of different activities, according to the following table 2 for supplementary method. The frequency and duration for each time were asked for each participant. Participants could choose one of the following frequency options, i.e., 1 ~ 2 times a week, 3 ~ 5 times a week, and > 5 times a week. Options including a range would be substituted by the midpoint of the range, e.g., we

assigned 1.5 times a week for the option “1-2times a week”. The duration for each time was also obtained by some options, i.e., < 30 minutes, 30-60 minutes or > 60 minutes. Each option would be substituted by the midpoint of the range, and those who chose over 60 minutes were substituted by 60 minutes. We calculated metabolic

equivalent times for each participant by adding time spent on each activity weighted by its metabolic equivalent score.

**Table 2 for supplementary method.** Reference for metabolic equivalent scores of different exercise types.

| **Physical activity** | **Metabolic equivalent scores** |
| --- | --- |
| Walking | 2.0 |
| Jogging | 7.0 |
| Swimming | 7.0 |
| Bicycling | 8.0 |
| Stair Climbing | 8.0 |
| Playing balls | 8.0 |
| Social dancing | 3.0 |
| Yoga | 2.5 |
| Aerobics | 4.0 |
| Strength Exercise | 8.0 |
| Mountain climbing | 8.0 |
| Others | 4.0 |

**Supplementary table 1**. Components of more recent dietary recommendations for blood lipid health*.

| **Diet complement** | **Intake goal** |
| --- | --- |
| Fruit | Consumption every day |
| Vegetable y | Consumption every day |
| Whole grains | Consumption every day |
| (Shell)fish | More than two times/week |
| Dairy | Consumption every day |
| Processed meats | ≤ Once a week |
| Sugar-sweetened beverages | No consumption |

*A healthy diet was defined as meeting at least half of the dietary recommendation (at least 4 items).

**Supplemental table 2**. Baseline characteristics of participants included or excluded from analyses due to missing information*.

| **Characteristics** |  | **Included participants (n=6617)** |  | **Excluded participants (n=876)** |  | ***p value*** |
| --- | --- | --- | --- | --- | --- | --- |
| Mean age (95%CI, years) |  | 36.5(36.3-36.7) |  | 38.2(37.6-38.8) |  | **<0.001** |
| Men |  | 1702 (25.7) |  | 405 (46.2) |  | **<0.001** |
| Married |  | 138 (2.1) |  | 19 (3.2) |  | 0.110 |
| Household income † |  |  |  |  |  | **0.019** |
| High |  | 491 (7.4) |  | 59 (9.9) |  |  |
| Medium |  | 5333 (80.6) |  | 482 (80.8) |  |  |
| Low |  | 793 (12.0) |  | 55 (9.2) |  |  |
| Education |  |  |  |  |  | 0.422 |
| College or above |  | 6373 (96.3) |  | 450 (95.1) |  |  |
| High School or equivalent |  | 195 (2.9) |  | 18 (3.8) |  |  |
| Less than high school |  | 49 (0.7) |  | 5 (1.0) |  |  |
| Occupational position |  |  |  |  |  | **<0.001** |
| High or above |  | 1103 (16.7) |  | 147 (24.7) |  |  |
| Medium |  | 2443 (36.9) |  | 225 (37.8) |  |  |
| Low |  | 3071 (46.4) |  | 223 (37.5) |  |  |
| Never smoking |  | 6092 (92.1) |  | 319 (89.6) |  | 0.097 |
| No heavy alcohol consumption |  | 6336 (95.8) |  | 350 (60.7) |  | **<0.001** |
| Top third LIPA |  | 2123 (32.1) |  | 235 (39.2) |  | **<0.001** |
| Healthy diet# |  | 2200 (33.2) |  | 198 (33.1) |  | 0.924 |
| BMI (mean, 95%CI,) |  | 23.3(23.1-23.4) |  | 23.8(23.4-24.1) |  | 0.035 |
| Self-reported comorbidities |  |  |  |  |  |  |
| Hypertension |  | 126 (1.9) |  | 38 (4.3) |  | **<0.001** |
| Diabetes |  | 51 (0.8) |  | 4 (0.5) |  | 0.400 |
| CVD |  | 23(0.4) |  | 4(0.3) |  | 1.000 |
| Cancer |  | 69(1.0) |  | 5(0.6) |  | 0.270 |
| Chronic bronchitis or COPD |  | 20(0.3) |  | 2(0.2) |  | 1.000 |
| Family history of diseases |  |  |  |  |  |  |
| Hypertension |  | 576 (8.7) |  | 50 (5.7) |  | **0.002** |
| Diabetes |  | 814 (12.3) |  | 71 (8.1) |  | **<0.001** |

BMI, body mass index; COPD, chronic obstructive pulmonary disease; CVD, cardiovascular disease; LTPA, leisure time physical activity.

* Continuous variables were expressed as mean (95% confidence interval), and categorical variables were expressed as number (percentage). P values were calculated using analysis of variance for continuous variables, and Pearson chi-squared test or Fisher's exact for categorical variables.

† Less than ￥50 000,￥50 000 to 299 999, and ￥300000 or more household income represented the high, medium, and low family income level, respectively.

# Healthy diet denoted ideal intakes of≥4 dietary components for cardiovascular health.

**Supplementary table 3.** Associations of SES with parameters of dyslipidemia and mediation effect of socioeconomic inequity in health attributed to lifestyle*.

| **Variables** | |  | **β** |  | **Standard error** |  | ***p value*** |
| --- | --- | --- | --- | --- | --- | --- | --- |
| TG |  |  |  |  |  |  |  |
| Unadjusted for lifestyle score | | | 0.058 |  | 0.03 |  | <0.001 |
| Adjusted for lifestyle score | | | 0.052 |  | 0.03 |  | <0.001 |
| Mediation effect* | | | 0.006 |  | 0.002 |  | 0.001 |
| TC |  |  |  |  |  |  |  |
| Unadjusted for lifestyle score | | | 0.011 |  | 0.037 |  | 0.452 |
| Adjusted for lifestyle score | | | 0.011 |  | 0.038 |  | 0.422 |
| Mediation effect* | | | -0.001 |  | 0.001 |  | 0.502 |
| LDL-C |  |  |  |  |  |  |  |
| Unadjusted for lifestyle score | | | 0.005 |  | 0.031 |  | 0.753 |
| Adjusted for lifestyle score | | | 0.006 |  | 0.031 |  | 0.679 |
| Mediation effect*) | | | -0.002 |  | 0.001 |  | 0.113 |
| HDL-C |  |  |  |  |  |  |  |
| Unadjusted for lifestyle score | | | -0.009 |  | 0.014 |  | 0.513 |
| Adjusted for lifestyle score | | | -0.008 |  | 0.014 |  | 0.536 |
| Mediation effect* | | | -0.0002 |  | 0.0005 |  | 0.663 |

SES, socioeconomic status; CI, confidence interval; TG, triglyceride; TC, total cholesterol; HDL-C, high-density lipoprotein cholesterol; LDL-C, low-density lipoprotein cholesterol; CI, confidence interval.

All models included age, sex, marital status, body mass index, self-reported comorbidities (including hypertension, diabetes, cardiovascular disease, cancer, and chronic bronchitis, emphysema, or chronic obstructive pulmonary disorder), and family history of diseases (including hypertension, diabetes, cardiovascular disease). Only the results comparing the low with high socioeconomic status are reported.

* Bias-corrected percentile method was presented based on 2,000 bootstraps samples.

**Supplementary table 4.** Associations of the healthy lifestyle score with incident dyslipidemia by socioeconomic status: sensitivity analyses *.

| **Analysis** |  | **Hazard ratio** |  | **95%CI** |  | ***p value*** |
| --- | --- | --- | --- | --- | --- | --- |
| Lifestyle score including sleep quality |  |  |  |  |  |  |
| High SES |  | 1(Reference) |  |  |  |  |
| Medium SES |  | 1.22 |  | 1.00-1.46 |  | 0.045 |
| Low SES |  | 1.33 |  | 1.01-1.74 |  | 0.041 |
| Excluding individuals with diabetes, CVD, cancer, chronic bronchitis, emphysema, or COPD at baseline | | | | | | |
| High SES |  | 1(Reference) |  |  |  |  |
| Medium SES |  | 1.20 |  | 0.99-1.46 |  | 0.064 |
| Low SES |  | 1.32 |  | 1.01-1.73 |  | 0.046 |

CI=confidence interval; CVD=cardiovascular disease; COPD=chronic obstructive pulmonary disease.

* All models included age, sex, marital status, body mass index, self-reported comorbidities (including hypertension, diabetes, cardiovascular disease, cancer, and chronic bronchitis, emphysema, or chronic obstructive pulmonary disorder), and family history of diseases (including hypertension, diabetes, cardiovascular disease). Only the results comparing the low with high socioeconomic status are reported.

**Supplementary Table 5.** Test of effect modification of age, gender and BMI on association between socioeconomic status and incident dyslipidemia^#^.

| Interactive terms† |  | Hazard ratio |  | 95%CI |  | *p-value* |
| --- | --- | --- | --- | --- | --- | --- |
| Age* SES |  | 1.01 |  | 1.00-1.02 |  | 0.028 |
| Gender*SES |  | 1.29 |  | 1.04-1.61 |  | 0.023 |
| BMI*SES |  | 1.02 |  | 1.00-1.04 |  | 0.026 |

CI: confidence interval; SES: Socioeconomic status.

† Multiplicative interaction was evaluated using hazard ratios for the interactive term between the age/gender BMI and SES.

# All models included age, sex, marital status, body mass index, self-reported comorbidities (including hypertension, diabetes, cardiovascular disease, cancer, and chronic bronchitis, emphysema, or chronic obstructive pulmonary disorder), and family history of diseases (including hypertension, diabetes, cardiovascular disease)

**Supplementary table 6.** Associations of socioeconomic status with incident dyslipidemia and mediation effect of socioeconomic inequity in health attributed to lifestyle: subgroup analyses*.

| **Subgroups** |  | **Hazard ratio** |  | **95%CI** |  | ***p value*** |
| --- | --- | --- | --- | --- | --- | --- |
| Men |  |  |  |  |  |  |
| Unadjusted for lifestyle score |  | 1.54 |  | 1.06-2.24 |  | 0.025 |
| Adjusted for lifestyle score |  | 1.48 |  | 1.01-2.16 |  | 0.043 |
| Mediation effect (β)* |  | 0.003 |  | -0.000-0.008 |  | 0.145 |
| Women |  |  |  |  |  |  |
| Unadjusted for lifestyle score |  | 1.25 |  | 0.84-1.85 |  | 0.271 |
| Adjusted for lifestyle score |  | 1.17 |  | 0.79-1.74 |  | 0.432 |
| Mediation effect (β)* |  | 0.001 |  | --0.0001-0.002 |  | 0.120 |
| 45 years or older |  |  |  |  |  |  |
| Unadjusted for lifestyle score |  | 1.55 |  | 1.04-2.32 |  | 0.032 |
| Adjusted for lifestyle score |  | 1.50 |  | 1.02-2.30 |  | 0.040 |
| Mediation effect* |  | 0.004 |  | -0.001-0.008 |  | 0.089 |
| Less than 45 years |  |  |  |  |  |  |
| Unadjusted for lifestyle score |  | 1.12 |  | 0.77-1.63 |  | 0.547 |
| Adjusted for lifestyle score |  | 1.09 |  | 0.75-158 |  | 0.649 |
| Mediation effect (β)* |  | -0.001 |  | -7.9--0.000-0.002 |  | 0.150 |
| Overweight or obesity |  |  |  |  |  |  |
| Unadjusted for lifestyle score |  | 1.58 |  | 1.03-2.41 |  | 0.035 |
| Adjusted for lifestyle score |  | 1.54 |  | 1.01-2.37 |  | 0.047 |
| Mediation effect (β)* |  | 0.002 |  | 0.0004-0.004 |  | 0.021 |
| Normal BMI |  |  |  |  |  |  |
| Unadjusted for lifestyle score |  | 1.00 |  | 0.06-16.88 |  | 0.999 |
| Adjusted for lifestyle score |  | 1.18 |  | 0.07-20.31 |  | 0.907 |
| Mediation effect (β)* |  | <0.001 |  | -0.002-0.002 |  | 0.965 |

CI, confidence interval; Overweight or obesity: BMI was greater than 25.0 kg/m^2^; Normal BMI: BMI ranged from 18.4 to 24.9 kg/m^2^.

* All models included age, sex, marital status, body mass index, self-reported comorbidities (including hypertension, diabetes, cardiovascular disease, cancer, and chronic bronchitis, emphysema, or chronic obstructive pulmonary disorder), and family history of diseases (including hypertension, diabetes, cardiovascular disease). Only the results comparing the low with high socioeconomic status are reported.

* Bias-corrected percentile method was presented based on 2,000 bootstraps samples.
